# TB and SIV Coinfection; a Model for Evaluating Vaccine Strategies against TB Reactivation in Asian Origin Cynomolgus Macaques: A Pilot Study Using BCG Vaccination

**DOI:** 10.3390/vaccines9090945

**Published:** 2021-08-25

**Authors:** Andrew D. White, Laura Sibley, Jennie Gullick, Charlotte Sarfas, Simon Clark, Zahra Fagrouch, Ernst Verschoor, Francisco J. Salguero, Mike Dennis, Sally Sharpe

**Affiliations:** 1 Public Health England, National Infections Service, Porton Down, Salisbury SP4 0JG, UK; Andrew.White@phe.gov.uk (A.D.W.); J.Gullick@soton.ac.uk (J.G.); Charlotte.Sarfas@phe.gov.uk (C.S.); Simon.Clark@phe.gov.uk (S.C.); Javier.Salguero@phe.gov.uk (F.J.S.); Mike.Dennis@phe.gov.uk (M.D.); Sally.Sharpe@phe.gov.uk (S.S.); 2 Department of Virology, Biomedical Primate Research Centre, Lange Kleiweg 161, 2288 GJ Rijswijk, The Netherlands; Fagrouch@bprc.nl (Z.F.); Verschoor@bprc.nl (E.V.)

**Keywords:** tuberculosis, SIV, macaques, coinfection, reactivation

## Abstract

This pilot study aimed to determine the utility of a cynomolgus macaque model of coinfection with simian immunodeficiency virus (SIV) for the assessment of vaccines designed to prevent reactivation of TB. Following infection caused by aerosol exposure to an ultralow dose of *Mycobacterium tuberculosis* (M. tb), data trends indicated that subsequent coinfection with SIVmac32H perturbed control of M. tb infection as evidenced by the increased occurrence of progressive disease in this group, higher levels of pathology and increased frequency of progressive tuberculous granulomas in the lung. BCG vaccination led to improved control of TB-induced disease and lower viral load in comparison to unvaccinated coinfected animals. The M. tb-specific IFNγ response after exposure to M. tb, previously shown to be associated with bacterial burden, was lower in the BCG-vaccinated group than in the unvaccinated groups. Levels of CD4+ and CD8+ T cells decreased in coinfected animals, with counts recovering more quickly in the BCG-vaccinated group. This pilot study provides proof of concept to support the use of the model for evaluation of interventions against reactivated/exacerbated TB caused by human immunodeficiency virus (HIV) infection.

## 1. Introduction

TB is one of the leading causes of human deaths from a single infectious agent worldwide. It is estimated that ten million people fell ill with tuberculosis (TB) in 2019, 8.2% of which were people coinfected with human immunodeficiency virus (HIV) [1]. During this period, TB was responsible for 1.4 million deaths, of which approximately 0.2 million deaths were among HIV-positive people [1]. Approximately 2 billion people are estimated to be infected with TB, and 5–10% of these individuals are at high risk of relapsing to infectious active disease during their lifetime [1]. Furthermore, this risk is believed to be increased for those also infected with HIV [2,3]. Therefore, the geographical overlap between HIV and TB epidemics, and the synergy between the pathogens, has the capacity to unlock a largely dormant reservoir of *Mycobacterium tuberculosis* (M. tb) contagion. TB disease is exacerbated following coinfection with HIV because the immune response to M. tb is dependent on CD4+ T-cells, which are the cells targeted and depleted by HIV. Activated Th1 effector T-cells drive the formation and development of granulomas in the lung, aiding with control of disease progression, thus factors that affect this population will also impact disease control.

Vaccination is the most effective way to control any infectious disease. The only vaccine currently available against TB, Bacillus Calmette–Guérin (BCG), is only partially effective against adult pulmonary TB [4]. Despite the concerted efforts focused on novel vaccine discovery, no new intervention has proven more effective than BCG in advanced clinical trials against initial TB infection. A variety of novel interventions, including postexposure vaccination against latent TB infection, are in development [5,6,7,8]. In the absence of verified correlates of protection, well-characterised animal models that accurately reflect human disease are essential to the development of new interventions [9]. Conventionally, most preclinical tuberculosis animal models focus on testing efficacy during the active phase of disease. Whilst small animal models of latent tuberculosis exist, there is concern that they may not adequately reflect human latent infection. Nonhuman primate (NHP) models of latent tuberculosis have also been developed and have confirmed that TB infection in the cynomolgus macaque can exhibit the characteristic features of latent stage disease [10]. Moreover, subsequent simian immunodeficiency virus (SIV) coinfection has been shown to induce reactivation of latent infection in this model [11]. However, the extended timescale required for the development of latent TB (approximately 8–10 months), and the likelihood that a significant proportion of animals will progress to active disease before latency is established, prohibits the use of this model in the rapid screening of novel interventions targeted at the prevention of reactivating TB disease.

In this pilot study, we used an ultra-low-dose (ULD) M. tb aerosol challenge system to establish a limited level of disease in Indonesian genotype cynomolgus macaques (ICM) over 16 weeks, then evaluated the impact of coinfection with SIV on disease burden over a further 16-week period characterised by quantifiable changes in pulmonary and extrapulmonary disease measurable by computer tomography (CT) and end-of-study pathology analysis [12]. The M. tb exposure dose selected for this study was informed by the findings of previous work that characterised the outcome of M. tb in cynomolgus macaques of the same origin [12], where it was shown to be sufficient to induce a consistent level of disease in all animals and low enough to resemble natural infection. The exposure dose was particularly important for this study, as the investigation of chronic infection and reactivation is dependent on the establishment of a controlled infection, which requires a long timeline and the ability to control disease. Other macaque populations, such as rhesus macaques or cynomolgus macaques of Mauritian genotype, are more susceptible to TB disease, and their intrinsic inability to control disease progression [13] would have made them unsuitable for this investigation. In addition, the capacity of the BCG vaccine to modulate SIV triggered progressive TB was assessed as an example of how the coinfection model could be applied for the evaluation of interventions to prevent destabilisation of chronic TB infection.

## 2. Materials and Methods

### 2.1. Experimental Animals

Nine male cynomolgus macaques (*Macaca fascicularis*) of approximately three years of age were obtained for the study from a characterised, closed, UK-based breeding colony, where genetic analysis previously confirmed the macaques to have the Indonesian genotype [14]. Compatible social groups were housed in accordance with Home Office (UK) [15] and NC3Rs guidelines [16] in cages with high-level observation balconies and extensive environmental stimulation and were provided with a wide range of dietary enrichment [17]. Animal procedures and study designs were approved by the Establishment Animal Welfare and Ethical Review Committee and authorised under a UK Home Office project license. Prior to challenge with M. tb, macaques were housed in cages approximately 2.5 m high by 4 m long by 2 m deep, constructed with high-level observation balconies and with a floor of deep litter to allow foraging. Following challenge, animals were transferred to banks of cages placed in directional airflow containment systems that allowed group housing and environmental control whilst providing a continuous, standardised inward flow of fully conditioned fresh air identical for all groups. Additional environmental enrichment was afforded by the provision of toys, swings, feeding puzzles and DVDs for visual stimulation. In addition to standard Old-World primate pellets, diet was supplemented with a selection of fresh vegetables and fruit.

For each procedure, the animals were sedated by intramuscular injection with ketamine hydrochloride (10 mg/kg) (Ketaset, Fort Dodge Animal Health Ltd., Southampton, UK). None of the animals had been used previously for experimental procedures, and each socially compatible group was randomly assigned to a study treatment. Prior to study enrolment, previous exposure to mycobacterial antigens was assessed using an IFNγ ELISPOT (MabTech, Nacka, Sweden) to detect responses to tuberculin-PPD (SSI, Copenhagen, Denmark), and pooled 15-mer peptides of ESAT6 and CFP10 (Peptide Protein Research LTD, Fareham, UK). The manuscript was prepared in accordance with the ARRIVE guidelines.

### 2.2. BCG Vaccination

**Figure 1:**
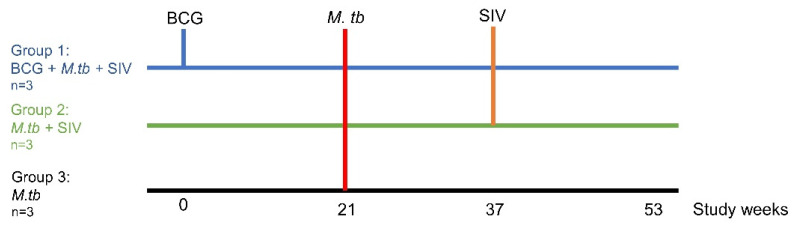
Study schedule.

The vaccination schedule relative to challenges with M. tb and SIV is shown in Figure 1. Three of the macaques were immunised by intradermal (ID) injection in the upper left arm with 100 μL of BCG vaccine, Danish strain 1331 (SSI, Copenhagen, Denmark), The BCG vaccine was prepared for intradermal administration according to the manufacturer’s instructions for administration to humans. One millilitre of Sauton’s diluent was added to a vial of BCG vaccine to give a suspension of BCG at an estimated concentration of between 2 and 8 × 10^6^ CFUs/mL. Vaccines were administered within one hour of vaccine reconstitution. Plate culture of residual vaccine onto Middlebrook 7H11 OADC agar confirmed the viability of the BCG, and the mean average administered dose was calculated to be 1.46 × 10^6^ CFUs.

### 2.3. Administration of M. tb Aerosol Challenge

Twenty-one weeks after vaccination, the group of BCG-vaccinated macaques (Group 1), together with six untreated macaques (Groups 2 and 3), was challenged by exposure to aerosols containing ultralow doses of M. tb Erdman strain KO1, as previously described [12]. A concentration of M. tb in the Collison nebuliser was selected to result in a retained dose of approximately five viable CFUs.

### 2.4. Administration of SIV Intravenous Challenge

Sixteen weeks after M. tb challenge, the three BCG-vaccinated animals, together with three of the unvaccinated animals, were challenged with 106–107 (TCID)_50_ SIVmac32H 11/88. Virus inoculum was diluted aseptically shortly before challenge and administered in 2 mL of RPMI + 1% FCS by inoculation into the femoral vein, as reported previously [18,19]. The dose of SIV delivered was confirmed by a dilution series of virus added to flasks of C8166 cells that were cultured and fed every 3–4 days until either cytopathic effect (CPE) was observed or 28 days had elapsed. The tissue culture infectious dose (TCID_50_) was calculated using the Kärber formula [20]. Infection was confirmed by culture of PBMCs isolated from challenged animals with C8166 cells and examining for cytopathic effect at the time of feeding (every 3–4 days), as described elsewhere [21].

### 2.5. Plasma RNA Load

Viral RNA was isolated using a QIAamp Viral RNA Mini Kit (Qiagen Benelux BV, Venlo, the Netherlands) following the manufacturer’s instructions. SIV viral loads in plasma were determined using a modified version of a published SIV-gag-based real-time PCR assay [22]. The SIV probe used was identical to the probe described by Leutenegger et al. [22] but using the quencher dye Black Hole Quencher 2. The forward (SIV31) and reverse (SIV41) primers were essentially identical to primers SIV.510f and SIV.592r35, with minor modifications to improve the sensitivity of the assay. The SIV31 and SIV41 primer sequences were 5’-CCAGGATTTCAGGCACTGTC-3’ and 5’-GCTTGATGGTCTCCCACACA-3’, respectively. The assay was carried out using the Brilliant^®^ II QRT-PCR Core Reagent Kit, 1-Step (Stratagene Europe, Amsterdam, the Netherlands) in a 25 µL volume with final concentrations of 160 nM for each primer, 200 nM for the probe, 5.5 nM MgCl2 and using 10 µL RNA. RNA was reverse transcribed for 30 min at 45 °C. Then, after a 10 min incubation step at 95 °C, the cDNA was amplified for 40 cycles, consisting of 15 s denaturation at 95 °C, followed by a 1 min annealing/extension step at 60 °C. All the reactions were carried out with an iQ™5 Multicolor Real-Time PCR Detection System (Bio-Rad Laboratories BV, Veenendaal, the Netherlands).

### 2.6. Postchallenge Clinical Assessment

Animals were sedated at two weekly intervals for clinical examination, including the measurement of body weight and temperature and blood sample collection with assessment of red blood cell (RBC) haemoglobin levels and erythrocyte sedimentation rate (ESR). RBC haemoglobin levels were monitored as an indication of anaemia, measured using a HemoCue haemoglobinometer (HemoCue Ltd., Dronfield, UK). ESR was monitored using the Sediplast system (Guest Medical, Edenbridge, UK) as a general measure of M. tb-induced inflammation. Animal behaviour was observed throughout the study for contraindicators, and the time of necropsy, if prior to the end of the planned study period, was determined by experienced primatology staff based on a combination of the following adverse indicators: depression or withdrawn behaviour, abnormal respiration (dyspnoea), loss of 20% of peak postchallenge weight, ESR levels elevated above normal (>20 mm), haemoglobin level below normal limits (<100 g/dL), increased temperature (>41 °C) and abnormal thoracic radiograph, which has been described previously [23].

### 2.7. Computed Tomography (CT) Imaging

CT scans were collected from sedated animals using a 16-slice Lightspeed CT scanner (General Electric Healthcare, Milwaukee, WI, USA) 6, 15, 18, 24 and 32 weeks after aerosol exposure to M. tb, as described previously [23]. Scans were evaluated by an expert thoracic radiologist blinded to the animal’s treatment and clinical status for the number and distribution across lung lobes of pulmonary lesions and the presence of extrapulmonary disease [17]. The disease burden attributable to infection with M. tb was scored using a relative scoring system based on the number of lesions present in lungs, spleen, liver, kidney and lymph nodes and the presence and extent of TB-induced structural abnormalities, as described previously [17].

### 2.8. Necropsy

The necropsies were conducted either when disease progressed to meet the criteria set as the humane endpoint, 16 weeks after SIV infection or 32 weeks after M. tb aerosol challenge. Animals were anaesthetised, and clinical data were collected. Blood samples were taken prior to euthanasia by intracardiac injection of a lethal dose of anaesthetic (Dolethal, Vétoquinol UK Ltd., Towcester, UK 140 mg/kg). A postmortem examination was performed immediately, and gross pathological changes were scored using an established system based on the number and extent of lesions present in the lungs, spleen, liver, kidney and lymph nodes, as described previously [24]. The lungs, including the heart and lung-associated lymph nodes, were removed intact. The lymph nodes were measured and examined for lesions.

### 2.9. Gross Pathology

The entire lungs were fixed by intra-tracheal infusion with 10% neutral-buffered formalin (NBF), as described elsewhere [23]. The lung lobes were sliced serially (~5 mm intervals). Discrete and coalesced lesions were counted and recorded, and the dimensions of the latter were measured. The presence of lung consolidation in each lobe was also recorded. A tissue slice containing obvious lesions (when present) was chosen from each lung lobe. Where gross lesions were not visible, a sample was taken from a predefined anatomical location from each lobe to establish consistency between animals. Sections of lung-associated lymph nodes (LALNP (those associated with the distal trachea and bifurcation)) and from extra-thoracic organs described above, were also fixed in buffered formalin.

### 2.10. Histopathology

All samples were processed to paraffin wax blocks using standard procedures sectioned at 4 µm and stained with haematoxylin and eosin (H&E). Slides were scanned digitally using the ‘3DHISTECH slide scanner and proprietary ‘Caseviewer’ software (both 3DHISTECH, Budapest, Hungary) used to capture, store and annotate digital images. All gross and histopathological examinations were carried out by qualified veterinary pathologists blinded to the treatment group. Each slide was evaluated for the presence of tuberculous lesions. The TB-associated lesions were recorded using the scoring system described previously [25,26]. Briefly, six different types of granulomas were identified. Types one (I), two (II) and three (III) were considered as ‘unorganised’ lesions, while types four (IV), five (V) and six (VI) were described as ‘organised’ granulomas. Type I was small, diffuse foci of macrophages and lymphocytes with scattered neutrophils and eosinophils, lacking clearly defined margins, infiltrate alveolar walls and extend into alveoli. Type II lesions were composed of similar cell types as type I but were larger in size and formed a more defined circumscribed focus of granulomatous inflammation, frequently circular and with variably demarcated border. Type III was as type II but with focal necrosis present, characterised by nuclear pyknosis and karyorrhexis with the loss of cellular architecture. Type IV was characteristically circumscribed granulomas consisting primarily of macrophages admixed with neutrophils and other leucocytes, with evidence of a few peripheral lymphocytes. Type V was organised lesions exhibiting necrotic foci with degenerated neutrophils, and type VI were classic, largely well-demarcated granulomas with central caseous necrosis and a variable rim of lymphocytes. For each lung tissue section, the total area of showing TB-associated lesions was calculated, and the total number of granulomas of each type were counted and recorded. Statistical analysis, X-sq for trend, was used to determine differences in the granuloma type distribution among groups.

### 2.11. Bacteriology

The spleen, kidneys, liver and tracheobronchial lymph nodes were sampled for the presence of viable M. tb postmortem, as described previously [27].

### 2.12. IFNγ ELISPOT

Peripheral blood mononuclear cells (PBMCs) were isolated from heparin-treated blood using standard methods. An IFNγ ELISpot assay was used to quantify the number of mycobacteria-specific IFNγ-producing T cells in PBMCs using a human/simian IFNγ kit (MabTech, Nacka, Sweden), as described previously [12,23,25]. In brief, 2 × 10^5^ PBMCs were cultured with 10 μg/mL PPD (SSI, Copenhagen, Denmark) or pools of overlapping 15-mer peptides spanning CFP10 or ESAT6 (Peptide Protein Research Ltd., Wickham, UK) in duplicate, or without antigen, in quadruplicate, and incubated for 18 h. Phorbol 12-myristate (Sigma-Aldrich Dorset, UK) (100 ng/mL) and ionomycin (CN Biosciences, Nottingham, UK) (1 μg/mL) were used as a positive control. After culture, spots were developed according to the manufacturer’s instructions. Plates were scanned and spots enumerated using a CTL Immunospot S6 reader and software (CTL, Germany). Determinations from replicate tests were averaged, and data were analysed by subtracting the mean number of spots in the medium-only control wells from the mean counts of spots in wells with antigen, or peptide pools, to derive an antigen-specific spot count. This value was multiplied by a factor of five and reported as IFNγ spot-forming unit (SFU) frequency per million PBMCs.

### 2.13. Whole-Blood Immunophenotyping Assay

Immunophenotyping was applied throughout the study to enable quantification of CD4+ and CD8+ T-cells that are known to decrease following SIV infection [28]. A 50 µL volume of whole heparinised blood was incubated with an antibody cocktail containing CD3-AF700, CD8-PeCy7, CD4-PE (all BD Biosciences, Oxford, UK) and a live/dead stain (Invitrogen, UK) for 30 min. The red blood cells were lysed using Utilyse as per the manufacturer’s instructions (Dako, Carpenteria, CA, USA) and incubated in the dark at room temperature (RT) for 1–3 h. Then, 8% formaldehyde was added to make a final concentration of 4% formaldehyde per sample and was stored in the dark prior to analysis. Before acquisition, 50 µL of Beckman Coulter Flow Count Fluorospheres (Beckman Coulter, UK) were added. Samples were run on the BD LSRII Fortessa (BD Biosciences, Oxford, UK). Data were analysed using FlowJo (version 9.7.6, Treestar, Ashland, OR, USA).

### 2.14. Statistical Analysis

Due to the small group sizes used in this pilot study, comparative statistical analysis was not applied to the data sets obtained.

## 3. Results

### 3.1. TB and SIV Disease Progression

**Figure 2:**
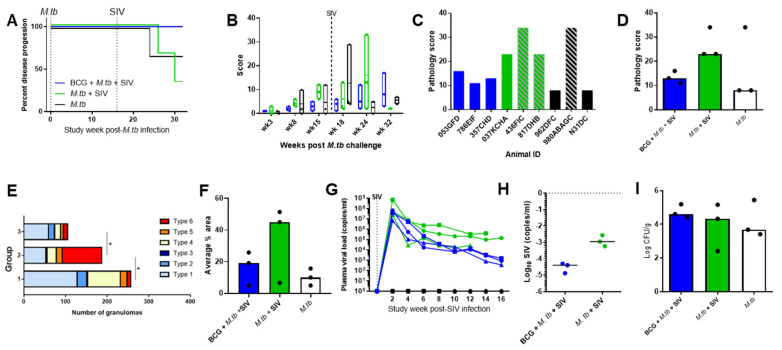
Measures of disease burden and progression in macaques following coinfection with M. tb + SIV or infection with M. tb alone. (**A**) Kaplan–Meier curve; (**B**) total score determined from CT scans; (**C**) combined gross pathology scores of all tissues measured, diagonal stripes indicate animals that were removed from the study early as disease progressed to meet humane endpoint criteria; (**D**) median combined gross pathology scores of the groups; (**E**) total number of granulomas (all types) in the lungs; (**F**) percentage of lung tissue covered with granulomas measured from microscopic analysis; (**G**) SIV viral load throughout the study; (**H**) median viral load measured in serum at postmortem for the SIV-infected groups; (**I**) median M. tb bacterial burden shown as the sum of CFUs/g cultured from all tissues analysed. Mann-Whitney U test performed on data set E, * denotes statistically significant difference *p* ≤ 0.05. Blue = Group 1: BCG + SIV + M. tb, green = Group 2: M. tb + SIV, black = Group 3: M. tb only. Median is shown for (**D**,**F**,**H**,**I**).

A presented dose of 19–26 colony-forming units (CFUs) of Erdman strain KO1 delivered by aerosol resulted in an estimated retained dose of four to six CFUs in the lungs of exposed macaques. After infection with M. tb alone, or M. tb and SIV, one animal in Group 1 (M. tb only) and two from Group 2 (M. tb + SIV) (Figure 2A) developed levels of disease that met humane endpoint criteria (as defined in Section 2). An indication for control of TB was seen in the BCG-vaccinated group, which received challenges with both M. tb and SIV, as all three animals controlled disease until the end of the study.

Disease burden was monitored at regular intervals throughout the study using CT scanning of the animals. Six weeks after M. tb infection, CT scores were highest in the animals in the M. tb-infection-only group in which disease progressed to meet humane endpoint criteria (Figure 2B). CT scores also increased in two of the unvaccinated coinfected animals after SIV infection as disease progressed to meet humane endpoint criteria at Week 23 after TB infection. One BCG-vaccinated coinfected animal developed increased nodule counts from Week 23 onwards, while lung nodule numbers below six were consistently recorded in the other two animals in the BCG group. Quantification of disease burden based on scores attributed for features visualised by CT scanning revealed a trend towards a lower burden in Group 1 (BCG + M. tb and SIV) that received a BCG vaccination than in unvaccinated animals (Groups 2 and 3) during the 16-week period after M. tb challenge. Similarly, CT scores were lower in the BCG-vaccinated group (Group 1) than in the unvaccinated coinfected animals (Group 2) in the first eight weeks after SIV challenge before disease progression required animals from Group 2 (M. tb + SIV) to be removed from the study.

Disease burden in tissues was quantified based on changes in gross pathology at the time of euthanasia either when humane endpoint criteria were met or at the planned conclusion of the study according to the PHE scoring system previously reported [13]. All animals showed evidence of TB disease (Figure 2C), with the highest overall pathology scores seen in Group 2 (M. tb + SIV) (Figure 2D). Lower pathology scores were seen in Group 1 (BCG-vaccinated + M. tb + SIV) than in Group 2 (M. tb + SIV).

Gross and microscopic lesions consistent with tuberculosis were observed in the lungs of all animals in all three groups (Table 1, Figure 2E). The number and the severity of disease varied in animals both within groups, as well as between groups.

Overall, severity of disease appeared greatest in Group 2 (M. tb + SIV), with a higher number of type 6 granulomas and a higher percentage of lung area made up of granulomatous tissue (Figure 2E,F). Animals 817DHB and 436FIC had 454 and 73 gross, discrete lesions, respectively, and adhesions were present between lobes. Furthermore, the remaining lobes in both animals were consolidated, with fulminating pneumonia, extensive caseation and airway invasion. A degree of peripheral fibrosis was seen in 817DHB, and calcification occurred in both animals. Disease was milder in the remaining animals, 037KCHA, with six gross discrete lesions, and infrequent, focal, type 5 granulomas with a degree of peripheral fibrosis. Systemic spread was evident in all three animals with lesions present in lung-associated lymph nodes (LALNs), kidney, spleen and liver.

In Group 1 (BCG + M. tb + SIV), gross lesion counts ranged from 3 to 73, with fibrous adhesions present in two animals. The size of the microscopic granulomas generally was larger than those observed in animals in Group 3 (M. tb only), and a degree of peripheral fibrosis was noted in all animals in this group. Airway invasion was noted in animal 357GHD only, but systemic spread was detected in all three animals. In Group 3 (M. tb only), gross lesion counts ranged between 1 and 222; gross, fibrous adhesions were noted in the animal with the highest counts (980ABAGC). Peripheral fibrosis was noted in two animals, whereas in the remaining animals, N31DC, lesions were too small for this change to be present. Systemic spread was detected in animals 962DFC and 980ABAGC only.

SIV viral load was determined using PCR applied to plasma collected from all groups at two weekly intervals following SIV infection (Figure 2G). Higher viral loads were seen in Group 2 (M. tb + SIV) compared to Group 1 (BCG + M. tb + SIV) (Figure 2H). SIV was not detected in Group 3 (M. tb only).

The bacterial burden was measured in representative samples of spleen, hilar lymph node, liver and right and left kidney, and the amount of M. tb in each tissue was calculated as colony-forming units per gram (CFUs/g) of tissue. Summation of CFUs determined from each of the tissues revealed the bacterial burden in all groups was similar, although the burden in both of the coinfected groups (Group 1 (BCG + M. tb + SIV) and Group 2 (M. tb + SIV)) showed a trend to be higher than that measured in Group 3 (M. tb only) (Figure 2I).

### 3.2. Immune Responses

**Figure 3:**
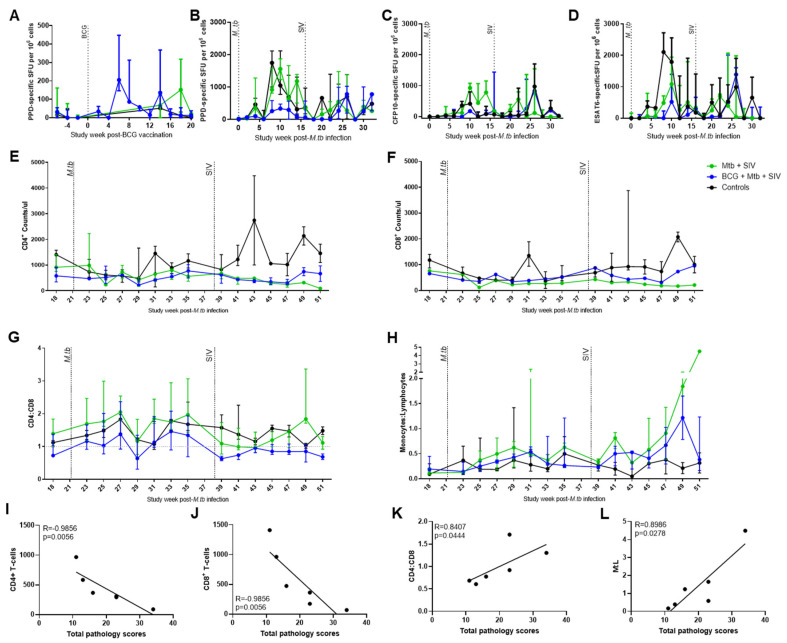
M. tb-specific IFNγ response measured by ELISPOT and CD4+ and CD8+ counts and CD4/CD8 ratio and M/L ratio determined by flow cytometry. (**A**) PPD-specific IFNγ ELISPOT SFU measured following BCG vaccination; (**B**) PPD-specific IFNγ ELISPOT SFU measured following M. tb and SIV infection; (**C**) ESAT6-specific IFNγ ELISPOT SFU measured following M. tb and SIV infection; (**D**) CFP10-specific IFNγ ELISPOT SFU measured following M. tb and SIV infection; (**E**) CD4+ cells/µL in whole blood following M. tb and SIV infection; (**F**) CD8+ cells/µL in whole blood following M. tb and SIV infection; (**G**) CD4/CD8 ratio determined in whole blood following M. tb and SIV infection; (**H**) M/L ratio defined in whole blood following M. tb and SIV infection. (**I**–**L**) Spearman’s Rank correlations of all SIV infected animals (Group 1 (M. tb + SIV) and Group 2 (BCG + M. tb + SIV)) p=0.05 (**I**) CD4+ T-cells correlation with total pathology scores, (**J**) CD8+ T-cells correlation with total pathology scores, (**K**) CD4:CD8 ratio correlation with total pathology scores, (**L**) M:L ratio correlation with total pathology scores. Blue = Group 1: BCG + SIV + M. tb, green = Group 2: SIV + M. tb, black = Group 3: M. tb only. Median and interquartile range shown.

The mycobacterial antigen-specific immune responses induced by BCG vaccination (Figure 3A) and M. tb challenge were measured every two weeks using an interferon gamma (IFNγ) ELISPOT assay containing the antigens: purified protein derivative (PPD) or peptides derived from M. tb early-secreted antigenic target 6 (ESAT6) or culture filtrate protein 10 (CFP10) (Figure 3B–D).

The frequency of PPD-specific IFNγ secreting cells increased in Group 1 animals (BCG + M. tb + SIV) and peaked six weeks after vaccination, then decreased to baseline levels by Week 20 (Figure 3B). Responses to ESAT6 and CFP10 were low in all of the groups prior to challenge with M. tb (Figure 3C,D).

During the sixteen-week period, after challenge with M. tb, the frequency of PPD-, ESAT6- and CFP10-specific IFNγ-secreting cells was notably higher in the unvaccinated macaques than in Groups 2 (M. tb and SIV) and 3 (M. tb), with differences being particularly evident after Week 8 (Figure 3B).

Following SIV challenge, trends were observed for the responses to all three antigens to increase, either between four to six weeks in Group 2 (M. tb + SIV) or between eight to ten weeks in Group 1 (BCG + M. tb + SIV). After Week 26, the response profiles in all groups showed similar trends, with a general decrease in M. tb-specific spot-forming units (SFUs) towards the end of the study.

The number of CD4+ and CD8+ T cells in whole blood was monitored using flow cytometry-based methods. After M. tb infection, all groups showed a decrease in CD4+ and CD8+ cell counts over the first four weeks after M. tb infection, which subsequently recovered over time (Figure 3E,F). SIV infection was followed by a consistent downward trend in CD4+ and CD8+ T-cell counts during the first ten weeks after infection in both Group 1 (BCG + M. tb + SIV) and Group 2 (M. tb + SIV). However, over the final six weeks of the study, CD4+ and CD8+ T-cell counts increased in Group 1 (BCG + M. tb + SIV), whereas numbers continued to decline in Group 2 (M. tb + SIV). Both CD4+ and CD8+ T-cell numbers at necropsy negatively correlated with pathology scores (both R = −0.9856, *p* = 0.0056) (Figure 3I,J). The CD4/CD8 ratio increased after M. tb challenge for all groups, decreased at Week 29, and increased again in a biphasic manner, and a high CD4/CD8 ratio correlated with higher pathology scores (Figure 3K). Following SIV infection, the CD4/CD8 ratio decreased in Groups 1 (BCG + M. tb + SIV) and 2 (M. tb + SIV) and remained lower in Group 1 (BCG + M. tb + SIV), whereas there was an increase in Group 2 (M. tb + SIV) (Figure 3G). CD4:CD8 ratio at necropsy correlated with pathology score (R = 0.8407, *p* = 0.0444) (Figure 3K). Monocyte/lymphocyte (M/L) ratio was also monitored throughout the study (Figure 3H). The levels were raised following M. tb infection, and intermittent increases in M/L were observed for individual animals in Group 3 (M. tb). An increase in M/L was observed in both Group 1 (BCG + M. tb + SIV) and Group 2 (M. tb + SIV) following SIV infection, particularly near the end of the study (Weeks 45–51). M/L in the two coinfected groups at their final time point, 16 weeks following SIV infection, correlated with a higher pathology score (R = 0.8986, *p* = 0.0278) (Figure 3L).

## 4. Discussion

Prevention of the reactivation of a latent TB infection is a huge challenge facing the control and eradication of TB, as the causes of reactivation are poorly understood, and there is the potential for infected persons to transmit disease within their community before the realisation that their infection has reactivated. Reactivation is particularly hard to model in macaques because TB disease is slow to manifest and consequently the models that currently exist need to run for a long period of time [11]. Furthermore, a spectrum of outcomes can occur following infection, from progressive disease to latency, and this lack of consistency in infection outcome reduces the practicality for testing novel interventions. The urgent need for reliable animal models of reactivation to enable an increased understanding of TB disease was highlighted by a meta-analysis of TB and BCG by the National Institute for Health Research (NIHR) [29]. Previous studies using the macaque model have demonstrated the potential for anti-TNF treatment or coinfection with SIV to reactivate latent TB [11,30]. We hypothesised that SIV coinfection in a host with an established, but controlled, TB infection would perturb immune control and exacerbate TB disease by simulating reactivation of infection. The potential to use a host with a well-controlled, low-level infection rather than one exhibiting clinical latency would significantly shorten the length of the study.

In this pilot study, we demonstrated that a well-controlled infection is established in cynomolgus macaques of Indonesian genotype following aerosol exposure to very low doses of aerosol delivered M. tb and is perturbed by subsequent infection with SIV. A higher proportion of the macaques in group 2, which received M. tb and SIV, developed a level of disease that progressed to meet humane endpoint criteria than was observed in animals infected with M. tb alone. The TB-related disease burden measured at the end of the study by pathology score and bacterial burden also suggested a greater level of TB disease in the coinfected group. Similarly, the presence of SIV infection concomitant with M. tb would explain the increased severity of disease observed in this group, likely resulting from a compromised adaptive immune response. Taken together, these observations would point towards a process akin to reactivation. To test this putative model of SIV co-infection-triggered reactivation as a potential platform for the assessment of novel interventions, BCG immunisation was used as a prototypical vaccine to explore the influence of a prophylactic vaccination regimen on measures of disease reactivation.

Even though the pilot study was limited by numbers of three per group, the differences between the groups are clear and repeated across various measures such that BCG appeared to confer some protection against M. tb and SIV coinfection. Following M. tb challenge, disease burden measured by CT score was lower in the BCG-vaccinated groups than in the unvaccinated groups, and similarly, during the first eight weeks after SIV infection when all coinfected animals remained in study, the disease burden was lower in the group that received BCG. After SIV infection, the viral load was initially similar in the BCG-vaccinated and unvaccinated groups but then decreased more quickly in the BCG-vaccinated group, resulting in a lower viral load at the end of the study, suggesting a BCG vaccine effect on the kinetics of viral control. The impact of BCG vaccination can also be seen by the improved control of M. tb-induced disease control such that all animals from this group completed the full study period.

Identification of differences between the groups based on histopathologic examination of tissues was challenging due to the large variation in disease severity in animals within each group. Type 6, caseating lesions, with additional features such as the presence of peripheral fibrosis, or foci of calcification, are often seen in more advanced disease stages, where there is an immune response to infection through ‘walling off’ of the granuloma with collagen, as well as dystrophic calcification of necrotic tissue. The BCG-vaccinated group, although it had higher granuloma scores overall, was predominantly of types 1 and 4, which are small, early lesions, whereas the unvaccinated coinfected group had more type 6 granulomas, and this, along with the lower area taken up with granulomas, suggests that the unvaccinated animals had more advanced disease and BCG-vaccinated animals may have been able to control the infection better.

Throughout the study, the ELISPOT assay was employed to measure the frequency of antigen-specific IFNγ-producing cells to provide an indicator of Th1 immune responses. An increase in PPD-specific IFNγ-secreting cells was observed in the animals in group 1 after BCG vaccination, although the response was lower and more short-lived than those observed after BCG vaccination in studies conducted with rhesus macaques [23,25,31]. However, the response profiles were similar to those we observed in cynomolgus macaques of Chinese origin, which were able to control a high-dose aerosol challenge of M. tb [32] and may point to other mechanisms of immune protection afforded by BCG in these cynomolgus macaque populations. Following M. tb infection, an increase in CFP10- and ESAT6-specific IFNγ-producing cells was measured in all animals. The frequency of responses to these diagnostic antigens is considered to be a biomarker reflective of disease progression, TB disease burden [33] and antigen load, with high frequencies associated with a poorer postchallenge outcome [24,25]. In the initial period following M. tb challenge and before infection with SIV, ESAT6 and CFP10 responses were lowest in the BCG-vaccinated animals, suggesting that TB disease was more controlled, in line with observations from efficacy assessments of BCG vaccination performed in rhesus [24,25] and cynomolgus macaques of Chinese origin [32]. The increase in frequency of M. tb-specific IFNγ-secreting cells measured following SIV infection is in line with Diedrich et al., who reported an increase after coinfection with SIV after a latent TB infection [11], thought to be related to reactivation of TB, reflecting a breakdown in control of TB infection. The increased level of response was delayed in the BCG-vaccinated group, which could indicate that control of disease was improved and took longer to break down. The lower level of CFP10 and ESAT6 responses and lower disease burden following infection with M. tb [12] in unvaccinated cynomolgus macaques of Indonesian origin in comparison to rhesus macaques has been observed previously and suggests a link between lower and potentially more controlled Th1 responses, leading to better control of TB disease. BCG vaccination in this study had the effect of reducing the CFP10 and ESAT6 response further, suggesting disease control after M. tb challenge and initially following SIV challenge, although coinfection then perturbed this control, as evidenced by the increase in M. tb antigen-specific IFNγ-secreting cell frequencies that corresponded to the higher disease burden measured in the vaccinated coinfected group compared to the M. tb only group.

The impact of SIV infection on the CD4+ and CD8+ T-cell population levels in peripheral blood was monitored using flow cytometry and revealed decreased T-cell counts in both the vaccinated and unvaccinated groups in line with previous reports in macaques [11]. A continuous decline in CD4+ and CD8+ T cells occurred during the 14 weeks following SIV infection in the M. tb and SIV coinfected group, whereas the decline ceased ten weeks after SIV infection in the BCG-vaccinated coinfected animals, with cell numbers recovering over the last six weeks of the study. A longer study would be required to determine whether a full recovery to pre-SIV infection levels might have occurred in BCG-vaccinated animals and explore further the influence of BCG vaccination on CD4 T-cell frequency, a vital component for immunological control of SIV infection.

M/L ratio has been identified as a potential risk factor for developing M. tb infection in HIV-negative [34] and HIV-positive persons [35] and has been observed to be higher in humans with active TB [36,37] and in those with HIV [38]. In this study, an increase in M/L was observed in some individuals at certain time points following M. tb infection, which could be interpreted as a sign of active disease that was subsequently controlled, but overall, M/L remained low for most of the M. tb infection phase of the study when data from other indicative measures used in the study suggested that the animals appeared to control disease relatively well. An increase in M/L was observed in the majority of the animals following coinfection, which could be related to reactivation of M. tb and also as a result of the SIV infection itself, as a high M/L ratio has been described in both disease states and provides a further measure and confirmation of the breakdown of M. tb disease control caused by coinfection with SIV.

In summary, this pilot study demonstrated that coinfection of macaques with SIV following an established ultra-low-level TB infection causes a worsening in TB-related disease and could potentially be used as a model of TB reactivation. The inclusion of a group that received vaccination with BCG prior to infection with either pathogen provided the opportunity to demonstrate the potential utility of this model for assessment of novel vaccine interventions as an improvement in TB disease was observed relative to the coinfection in an unvaccinated control group. Clinical trials involving vaccination of interferon gamma release assay (IGRA) positive people are underway [5], demonstrating the requirement for vaccines for deployment in those potentially infected with TB and highlighting the need for relevant models to enable the assessment of vaccine efficacy under these conditions. Further work involving larger group sizes is required to refine and extend the characterisation of the model.

## Figures and Tables

**Table 1:** Gross pathology observed in histopathology lung tissue sections.

	Animal ID	Discrete Lesions	Coalescing	Consolidation
Group 1: BCG + M. tb + SIV	053GFD	3	0	0
	768EIF	27	0	0
	357GHD	73	0	0
Group 2: M. tb + SIV	037KCHA	6	0	0
	436FIC	73	1	3
	817DHB	454	0	3
Group 3: M. tb	962DFC	4	0	0
	980ABAGC	222	0	0
	N31DC	1	0	0

## Data Availability

All data generated or analysed during this study are included in this published article.
